# The Impact of a New Pedagogical Intervention on Nursing Students' Knowledge Acquisition in Simulation-Based Learning: A Quasi-Experimental Study

**DOI:** 10.1155/2018/7437386

**Published:** 2018-10-01

**Authors:** Thor Arne Haukedal, Inger Åse Reierson, Hanne Hedeman, Ida Torunn Bjørk

**Affiliations:** ^1^Department of Nursing and Health Sciences, University of South-Eastern Norway, Post Box 235, 3603 Kongsberg, Norway; ^2^Department of Nursing Science, University of Oslo, Post Box 1130, Blindern, 0318 Oslo, Norway

## Abstract

Simulation-based learning is an effective technique for teaching nursing students' skills and knowledge related to patient deterioration. This study examined students' acquisition of theoretical knowledge about symptoms, pathophysiology, and nursing actions after implementing an educational intervention during simulation-based learning. A quasi-experimental study compared theoretical knowledge among two groups of students before and after implementation of the intervention. The intervention introduced the following new components to the existing technique: a knowledge test prior to the simulation, video-recording of the performance, and introduction of a structured observation form used by students and facilitator during observation and debriefing. The intervention group had significantly higher scores on a knowledge test conducted after the simulations in comparison to the scores in the control group. In both groups scores were highest on knowledge of symptoms and lowest on knowledge of pathophysiology; the intervention group had significantly higher scores than the control group on both topics. Students' theoretical knowledge of patient deterioration may be enhanced by improving the students' prerequisites for learning and by strengthening debriefing after simulation.

## 1. Introduction

Simulation-based learning (SBL) is a technique [1] widely used in nursing education, the use of which as an educational tool to achieve a wide range of learning outcomes has been supported by a multitude of studies [2]. One important outcome of nursing education is improved recognition and management of patient deterioration; these are essential nursing skills that students should begin to develop while in school [3], and students need a wide range of knowledge to recognize and act upon the signs of deterioration.

The relationship between theory and practice is a complex challenge in professional education. This is widely documented and commonly termed as a “gap” [4]. To reduce this gap, theoretical knowledge and practical experience must be integrated. SBL is a pedagogical approach that can be considered a “third learning space” between coursework and practicums; this approach may bring the content and process of theoretical work and practical training closer to each other [5].

SBL has been said to be a more effective teaching strategy than classroom teaching for the development of assessment skills for the care of patients with deteriorating conditions [6]. Simulation provides an opportunity to be exposed to critical scenarios and can highlight the clinical signs and symptoms the students will have to deal with in these situations [7]. It provides inexperienced students the opportunity to use their knowledge in a simulated situation, which mirrors the clinical context without the risk of harming actual patients [8]. Situated learning theory claims that learning is influenced by the context in which it occurs [9]. Tun, Alinier, Tang, and Kneebone [10] argue that the aspect of fidelity may hinge on the learners perceived realism of the context and that a simulation may seem realistic to students who lack experience. Lavoie, Pepin, and Cossette [11] call for educational interventions that can enhance nursing students' ability to recognize signs and symptoms in patient deterioration situations.

Many studies claim that SBL may improve theoretical knowledge acquisition [12–16]. A review by Foronda, Liu, and Bauman [17] suggested that simulation was an effective andragogical method for teaching skills and knowledge and called for more research to strengthen the evidence related to what types of nursing knowledge and nursing content could be effectively developed through SBL.

A review of empirical studies of educational interventions related to deteriorating patient situations showed that few used objective assessment as an outcome measure: only just over one-third measured improvement in knowledge, skills, and/or technical performance [18]. On the other hand, a plethora of studies are concerned with the students' experiences during SBL; these studies have found that students generally show a high degree of satisfaction with SBL as an educational technique because they often experience increased knowledge and confidence [3, 19, 20]. At the same time, however, there is a broad tendency for nursing students to overestimate their skills and knowledge in self-reports [21]. An essential component of quality assurance in nursing education thus remains to evaluate students' knowledge acquisition [22].

The present study compared acquisition of theoretical knowledge by two cohorts of nursing students in the course of six simulated scenarios on patient deterioration before and after the implementation of an educational intervention during simulation-based learning. The intervention aimed to improve students' learning prerequisites and strengthen debriefing in simulation. The aim of the study was to explore whether there was a difference in students' knowledge level before and after the educational intervention. The following research questions were developed:

- What are the differences in posttest scores on the knowledge test between the control group and the intervention group?
- What are the differences between stimulus (pre-) and posttest scores in the intervention group?

## 2. Methods

### 2.1. Intervention

In this study, we present an intervention to enhance students' theoretical knowledge via simulation-based learning and measure this development using an objective assessment. The intervention was inspired by the First2Act model, as described by Buykx and colleagues [3, 20]. First2Act was developed to improve nurses' emergency management skills [20], and it comprises five components: developing core knowledge, assessment, simulation, reflective review, and performance feedback. These components were set on the basis of experiential learning theory and empirical pedagogical literature (e.g., [23, 24]). A lack of theory-based research in simulation hampers the development of coherence and external validity in this field of research [25]. The present study uses First2Act as an explicit theoretical framework. Its distinct components are pedagogically founded and are hypothesized to enhance learning in advanced simulation [20]. Due to the importance of feedback in simulation-based learning [26], the feedback processes were enhanced beyond those introduced by First2Act [20].

The simulations were conducted before the students' first clinical practicum in hospital medical or surgical units. Simulation training before a practicum can, if the experiences reflect the way knowledge and actions will be used in actual practice, provide the students with authentic activities that mirror the forthcoming experiences in the real world of nursing [9]. In both cohorts, the students participated in a total of six scenarios where the patient developed a deteriorating condition, respectively, angina pectoris, cardiac arrest, hypoglycemia, postoperative bleeding, worsening of obstructive lung disease, and ileus. The scenarios were inspired by scenarios already created by the National League for Nursing and Laerdal, a medical company (Laerdal Medical Corporation 2008), and refined in collaboration with practicing nurses to suit a Norwegian context. The scenarios were carried out over two days, meaning that the students were given the repeated opportunity to collaborate on the assessment and treatment of deteriorating patients and to repeatedly go through the cycles of reflection before, in, and on action [27]. The students were organized into previously established learning groups each consisting of 5–9 students. Students in both cohorts had completed theoretical education on pathology, nursing subjects, and basic life support and had learned a variety of practical nursing skills in the simulation center.

Two students acted as nurses in each scenario, with one as the leading nurse; the remaining students were observers or next-of-kin. All students acted the role of leading nurse at least once during simulation and most twice. The students received a short synopsis of the six scenarios one week before the simulation to give them the opportunity to prepare for the simulations. During simulation, one of the faculty members had the role of facilitator, while another operated the manikin VitalSim (Laerdal Medical, Norway).  Table 1 details the structure of the scenario simulation in both cohorts participating in the study.

Our intervention introduced the following new components to the existing procedure.

(1) A knowledge test with multiple-choice questions (MCQs) was conducted one week before simulation as a stimulus for learning, to give the students the opportunity to prepare in advance. The questions covered core knowledge associated with each of the scenarios; students received individual electronic feedback giving the correct answers.

(2) The simulation performance was video-recorded on an iPad (Table 1).

(3) While observers and facilitators in the 2013 cohort gave feedback in relation to general learning outcomes, a structured observation form was developed for the 2014 cohort that covered scenario-specific observations and actions (Table 1), for example, measuring blood pressure, correct medication administration, when to call for help, and priority of actions.

(4) The debriefing was divided into two sessions: First, the students who had performed the simulation watched the video-recording, allowing for an assessment of their own performance (Table 1). Meanwhile, the observers planned and discussed the feedback they would provide the performing students, with reference to the structured observation form, and the facilitator and operator did likewise. The observation form described correct nursing actions and observations related to scenario-specific learning outcomes. Second, a facilitator-led debriefing was conducted following the framework described in Debriefing Assessment for Simulation in healthcare (DASH)® [28]. The new observation form also guided the facilitators during debriefing.

### 2.2. Study Design

This study used a two-group quasi-experimental design [29] that compared students' knowledge acquisition between a control and an intervention group. The control group experienced simulated scenarios according to their existing study program, while the intervention group experienced simulated scenarios based on a new pedagogical design, implemented one year later.

### 2.3. Sample and Setting

All students in the second year of their bachelor's degree in nursing at a university college in Norway were invited to participate in the study in 2013 (99 students) and in 2014 (91 students). The students were informed about the study in class and on the institution's digital learning platform. In December 2013, the 68 students who agreed to join the study participated in the scenario simulations as the control group; of these, 60 agreed to participate in the posttest. In December 2014, the 69 students who agreed to join took part in the scenario simulations as the intervention group; of these, 53 agreed to participate in the posttest. Of these 53 students, 40 participated in the stimulus test conducted before the simulation.

### 2.4. Development of the Instrument

The instrument had two sections. The first section included demographic data: age, gender, if they had worked in the health service and if so how long, and if they had experiences with simulation. The second section consisted of a multiple-choice questionnaire (MCQ) developed to function as the stimulus test as well as the posttest (Table 2).

The questionnaire consisted of 18 questions related to the deterioration of the patient's condition, 3 from each of the 6 different scenarios that students simulated. Six questions covered pathophysiology, for example, “Which situations can lead to hypovolemia?”; five questions covered symptoms, for example, “What are the two symptoms that can be present during an attack of angina pectoris?”; five questions covered nursing actions, for example “How do you handle an unconscious diabetic patient?”; and two questions covered prioritization of nursing actions, for example, “Range in prioritized order actions with a patient with cardiac arrest.” Of the 18 questions, 14 had 4 answer options, where students should mark off 2 correct answers; 2 had 3 answer options, where students should mark off 1 correct answer; and in the last 2 questions students were required to rank 4 answer options. The MCQ was developed by the facilitators, and content validity was established by experienced practicing nurses. The instrument had medium internal consistency, with Cronbach's alpha of 0.62 for the control group, 0.73 for the intervention group, and 0.62 for the stimulus test. These somewhat low numbers may be because the instrument focused on multiple content areas but had only 18 questions.

### 2.5. Data Collection and Analysis

Both posttests were completed as paper-and-pencil tests on a written form immediately after the last scenario simulation. This format was chosen to achieve the highest possible response rate [30]. The stimulus test for the intervention group was completed electronically through a digital learning platform one week before the SBL started.

Data analysis was performed with SPSS, version 22. Homogeneity between the groups was tested with descriptive summary statistics; then, knowledge scores were analyzed with descriptive statistics, and comparisons between the control and intervention groups were conducted with independent-samples t-test. A paired-sample t-test was used to assess difference between knowledge scores from the stimulus test and posttest mean scores in the intervention group. Effect size was computed using Cohen's d [31]. Age-related differences in scores were analyzed with analysis of variance (ANOVA).

### 2.6. Ethical Considerations

The study was approved by the university college where it was conducted and the Norwegian Social Science Data Services (project number 36135). Return of the questionnaire was considered to constitute consent to participate.

## 3. Results

Participant characteristics are shown in Table 3.

There was homogeneity on all tested characteristics between the two groups of students who participated in the posttest, with the exception of years of work experience (Table 3). We did not control for the differences in years of work experience at baseline.

Mean scores and standard deviations were calculated among all students who completed the knowledge test. There was a significant improvement in posttest knowledge scores between the control group (M=8.9 SD=3.2) and the intervention group (M=11.2 SD=3.5), p< 0.001. Effect size was d=0.68, considered a medium-sized effect [22].

The participants were divided into three age categories. Mean knowledge score for participants <22 years old in the control group was 8.5 (n=37) and in the intervention group 10.5 (n=31); participants aged 22–26 years had mean score of 9.1 (n=15) in the control group and 11.8 (n=8) in the intervention group; and mean score for participants >26 years was 10.6 (n=8) in the control group and 12.4 (n=14) in the intervention group. These differences were not statistically significant.

**Figure 1:**
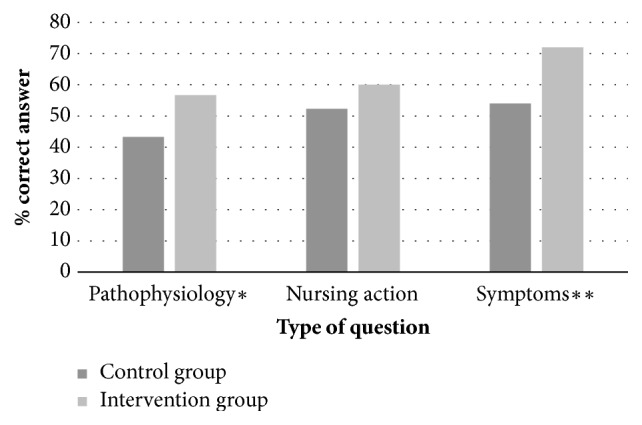
Posttest knowledge scores before and after the intervention. *∗*p=0.001 *∗∗*p<0.001.

The intervention group had significantly higher scores than the control group on questions concerning pathophysiology knowledge (p=0.001) and knowledge of symptoms (p<0.001) (Figure 1). Within both groups, questions concerning pathophysiology had lower scores than questions about symptoms and nursing actions.

Forty students in the intervention group completed both the stimulus test and the posttest; their scores were significantly higher on the posttest than on the stimulus test (M=12.0 SD=3.2 versus M=8.9 SD=3.1), p< 0.001. Effect size was d=1.1, considered as a large-sized effect [29].

## 4. Discussion

The purpose of the study was to examine the role of the educational intervention in the students' acquisition of knowledge. The knowledge test results show significantly greater improvement in posttest scores in the intervention group than in the control group. One component of the intervention was the introduction of a stimulus test, and because we wanted to investigate the effect of this component, no such test was given to the control group. It was found that there was a significant improvement in posttest scores compared with scores on the stimulus test. In the following, we will discuss the specific elements of the intervention that may have impacted the difference in the results between the two groups.

First, introduction of a knowledge test before the scenario simulation may stimulate students to strengthen their cognitive learning and is one aspect of participant preparation as described in the International Nursing Association for Clinical Simulation and Learning (INACSL) “Standards of Best Practice: Simulation^SM^” [32]. This is because a stimulus for learning can make the students more aware of both the knowledge they have and the knowledge they lack. It can thus serve as a “repetition trigger” prompting students to brush up on relevant topics such as pathophysiology as a preparation for simulation exercises. Baseline theoretical knowledge is necessary to acquire competence acquisition through SBL [33]. Similarly, Flood and Higbie [34] found that a relevant didactic lecture could be useful to strengthen cognitive learning on blood transfusion. To discover one's own lack of knowledge in a test conducted prior to the simulation may encourage more preparation and may furthermore increase students' attention during simulation and debriefing because they recognize the relevance of the test content as they practice and reflect. This strengthened knowledge base gives more substance to students' reflections and problem analysis during debriefing and thereby improves their knowledge development [23].

Viewing of video-recordings by students in the roles of nurses was the second component of the intervention that we see as potentially helpful. Video-assisted debriefing led by a facilitator is often used in SBL, although its effect is uncertain [35]. By completing the scenario and then watching their own video-recorded performance immediately afterward, the students who had acted as nurses got an opportunity to assess themselves; similar to the stimulus test, self-assessment can make students aware of both the knowledge they possess and the knowledge they lack to help them perform necessary actions. Video-based self-assessment in particular can help students develop awareness of their strengths and weaknesses [36]. However, it has been reported that some students “felt ashamed” when watching themselves onscreen [37]; to preclude this, we decided that our participants should view their performance alone, without interference of teacher and peers, so as to focus on learning, not on the judgment from others. Thereby, the students who had acted in the scenario were also given the opportunity to gain the observer's perspective. We expected that this would have reduced stress and thereby provided the opportunity for improved preparation before debriefing, also leaving them readier to focus on knowledge development together with their peers during the debriefing session.

The third new element of the intervention was the structured observation form, which focused on specific actions in each scenario. In this study, both the observers and the facilitator used the same observation form, which may have contributed to a clear focus on knowledge of the signs of deteriorating conditions; when students respond appropriately and then verbalize their deliberations, knowledge application is taken to be demonstrated [33]. An observation form functions as a tool that mediates learning [38, 39] and draws students' attention to the importance of change in patients' condition. The use of observation tools has been reported to engage the observer in learning [40–42] and facilitate observational learning by focusing on important aspects [43–45]. The observation form may have triggered assessments of actions based on specific professional knowledge rather than an overall approach.

A schedule of six scenarios in the course of two days afforded the students the chance for repetitive practice of important actions involved in handling deteriorating patients. Although the scenarios were different, the focus remained on key observations and actions to counteract deterioration, allowing ample practice for observing and handling common events such as low blood pressure or oxygen deficiency. Repetition is recommended as a best practice in learning [46]. Marton [47] argues that students need to be exposed not only to similarities but also to differences, in order to connect knowledge to different situations. The observation form highlighted key observations and may have helped the students to verbalize these aspects, make them explicit, and thereby promote transfer of knowledge from one situation to another. The students were exposed to many variations through the scenarios, and this may have improved their knowledge about symptoms, explaining why they had the highest score on symptoms.

Students' knowledge scores increased with age, in both groups. The finding was not significant but could indicate a trend. Though Shinnick, Woo, and Evangelista [48] claim that age is not a predictor of knowledge gain, increasing age may nevertheless indicate greater beneficial experience; thus, the stimulus test may be of even greater importance to younger students as a stimulus to learning—perhaps especially during SBL, for which baseline theoretical knowledge is one of several necessary antecedents [3, 33].

Both groups had the highest scores on knowledge of symptoms, lower on appropriate nursing actions, and lowest on knowledge of pathophysiology. We can only speculate with regard to this finding that it may be easier to acquire knowledge about symptoms and actions because this type of knowledge can be enhanced through visualization—by handling the actual symptoms of deteriorating patients, watching themselves on video, and taking the observer role. The use of manikins can be advantageous in this regard because symptoms can be portrayed via manikin's software, which can increase student's attention to the symptoms. It is also possible that pathophysiology requires a deeper understanding, meaning that it involves knowledge as justification for action. Recognizing symptoms in time is an important part of identifying signs of deteriorating conditions [12], and therefore high scores on knowledge of symptoms are a valuable finding. The intervention group had significantly higher scores than the control group on knowledge of pathophysiology and symptoms. This indicates that the new components of stimulus test, video viewing, and observer forms positively influenced the students' acquisition of knowledge. Both groups of students had limited clinical experience at this point in their education, which may explain why they did not achieve higher scores in general.

## 5. Limitations of the Study

The findings of this study may be of interest to educators because how to enhance students' knowledge acquisition is an increasingly important issue in SBL. The results of this study, however, are based on only a small sample recruited from only one school of nursing, which limits their generalizability. We used a convenience sample in this study, and the intervention group had 1.1 years more work experience than the control group. It is possible, though difficult to gauge, if levels of work skills could influence these students' overall scores. However, there was no significant difference in the two student groups' experience with critically ill patients.

Although the use of MCQs is a common approach in knowledge assessment, there are discussions about whether they really fit the purpose [49, 50]. Here, because stimulus test and posttest consists of the same questions, we are aware that students may remember correct answers from the stimulus test and therefore score higher on the posttest. This may mean that students have primarily gained knowledge from the stimulus test and not the other components of the intervention. Nevertheless, the significant increase in scores between the stimulus test and posttest in the intervention group suggests that the other components also are decisive in the students' knowledge acquisition. In addition, knowledge was measured only one time after the simulation, thus yielding no information on long-term knowledge retention. Finally, correct answers on MCQs do not necessarily correspond with students' actions in real situations of patient deterioration.

## 6. Conclusion

Students' knowledge scores were compared before and after an educational intervention during SBL. The results showed significantly greater improvement of scores in the intervention group than in the control group. Based on these findings, we assume that pedagogical underpinning of SBL, which emphasizes improvement of students' prerequisites for learning and strengthens the debriefing, can positively influence students' knowledge acquisition.

## Figures and Tables

**Table 1:** Structure of the scenario simulation for both cohorts.

	2013 cohort Original scenario simulation	2014 cohort Intervention
Briefing	15 minutes	15 minutes
Simulation	10 minutes Students observed in accordance with overall learning outcomes	10 minutes Video-recording of nurse actors Observation according to a structured observation form
Debriefing	20 minutes Facilitator led debriefing. Learning outcomes provided the basis for reflection	Session 1 15 minutes Viewing of video-recording by nurse actors Observers and facilitator/operator discussed observation and planned their feedback Session 2 20 min Facilitator led feedback according to the checklist

**Table 2:** Multiple-choice questionnaire.

	**Multiple choice questions – tick off the alternatives (the required number) you consider correct.**
	**Theme: Angina pectoris**
1	How does Nitroglycerin (NG) work? Tick off the two alternatives you consider to be correct.
	(i) Reduces the transfer of pain in the nervous system
	(ii) Reduces venous flow to the heart
	(iii) Improves the level of oxygen (pO2) in the blood
	(iv) Can trigger dizziness and fall in blood pressure
2	What are the two symptoms that can be present during an attack of angina pectoris? Tick off the two alternatives you consider correct.
	(i) Chest pain decreases after intake of NG
	(ii) The patient's lips turn cyanotic
	(iii) Frequence of pulse and blood pressure will decrease
	(iv) The patient may become winded/breathless during exertion
3	Range in prioritized order the actions you would perform with a patient admitted to hospital with angina pectoris (1 most important – 4 least important)
	(i) Administration of oxygen
	(ii) Insertion of peripheral vein cannula
	(iii) Blood sampling and ECG
	(iv) Administration of nitroglycerin

	**Theme: Cardiac arrest**
4	Which statements are correct? Tick off two alternatives.
	(i) The most common cause of cardiac arrest is acute heart infarction
	(ii) Cardiac arrest implies that the infarction is large
	(iii) Resuscitation is effective whether it starts at once or after a few minutes
	(iv) Abnormal breathing in an unconscious patient indicates cardiac arrest
5	Which two statements about heart compression are correct?
	(i) Depth of compression should be 5-6 cm
	(ii) Number of compressions should be at least 100/minute
	(iii) The most important to prioritize the first minutes after cardiac arrest is effective heart compression
	(iv) Resuscitation should always start with 30 compressions in a row
6	Range in prioritized order actions with a patient with cardiac arrest
	(i) Alert others (call)
	(ii) Heart compression
	(iii) Ventilation
	(iv) Check if the patient has gotten a pulse again

	**Theme: Hypovolemia/bleeding**
7	Which two statements are correct concerning bleeding and blood transfusion?
	(i) Blood transfusion is required if hgb falls 20%
	(ii) Bleeding through the bandage after a hip operation indicates a large loss of blood
	(iii) Reactions to a transfusion may normally occur the first 15 minutes after the transfusion has started
	(iv) Blood transfusion is normally required when hgb-values < 7g/100ml
8	What are the symptoms of blood loss/development of shock? Tick off two alternatives you consider correct.
	(i) Low blood pressure (<90 mmHg)
	(ii) Warm and red skin color
	(iii) Slow and irregular pulse
	(iv) Increasing apathy/confusion
9	Tick off two actions you consider most important to prioritize with a patient developing shock
	(i) Administration of oxygen
	(ii) Insert a urinary catheter
	(iii) Intravenous hydration
	(iv) Raise head-end of bed to ease ventilation/respiration

	**Theme: COPD**
10	What is the meaning of a COPD patient's habitual spO2 values? Tick off/mark the alternative that is correct (one tick).
	(i) The patient's spO2 values during the best phase of the disease
	(ii) The patient's spO2 values during worsening of COPD
	(iii) The patient's spO2 values with COPD grade 3 or 4
11	Which symptoms are typical during acute worsening of COPD? Tick off two alternatives you consider correct.
	(i) Restless and anxious patient
	(ii) Low values of O2 (spO2) and Co2 (spCo2)
	(iii) Inspirational stridor (difficult to breathe in)
	(iv) Expirational stridor (difficult to breathe out)
12	Tick off two actions you consider most important to prioritize with a patient with worsening COPD
	(i) Administer prescribed medications
	(ii) Abundant administration of oxygen
	(iii) Create a calming environment
	(iv) Measure O2 and CO2 in blood sample before treatment begins

	**Theme: Diabetes/hypoglycemia**
13	Which keywords match type-1 diabetes? Tick off two alternatives you consider correct.
	(i) Auto-immune disease
	(ii) Non-existent production of insulin
	(iii) Insulin resistance
	(iv) Part loss of insulin production
14	What are the symptoms in a patient with a mild degree of hypoglycemia? Tick off two alternatives you consider correct.
	(i) Loss of consciousness
	(ii) Hunger
	(iii) Diplopia
	(iv) Shivering
15	How do you handle an unconscious diabetic patient? Tick off one alternative.
	(i) As if the patient had hypoglycemia (give sugar)
	(ii) As if the patient had hyperglycemia (give insulin)
	(iii) Never treat the patient before you know the values of sugar in the blood

	**Theme: Ileus/hypovolemia**
16	Which situations can lead to hypovolemia? Tick off two alternatives you consider correct.
	(i) Cancer in the bowels
	(ii) The normal passage of intestinal content has stopped
	(iii) Paralysis of the bowels
	(iv) Feces leaks into the abdominal cavity
17	Which symptoms may be present during hypovolemia? Tick off two alternatives you consider correct.
	(i) Extended abdomen or dry mucous membranes
	(ii) Standing skin folds
	(iii) Abundant light-colored urine
	(iv) High blood pressure
18	Which two actions are the most important when one suspects that a patient has ileus?
	(i) Administer pain medication
	(ii) Administer a laxative
	(iii) Aspiration of ventricular content
	(iv) Careful stimulation of the bowels with soup

**Table 3:** Characteristics of the participants.

	Control	Intervention	t-test/*χ*^2^
	n = 60	n = 53	p-values
Age (M ± SD)	23.5 ± 5.6	24.5 ± 6.7	0.355^a^
Gender			
Female (n %)	55 91.7	50 94.3	*χ* ^2^ 0.306
Male (n %)	5 8.3	3 5.7	0.584
Work experience in health care before starting the nursing bachelor			
Yes (n %)	47 78.3	45 84.9	*χ* ^2^ 0.803
No (n %)	13 21.7	8 15.1	0.370
Years of work experience in health care			
(M, ± SD)	1.3 ± 1.5	2.4 ± 3.7	0.038^a^
Experience with critically ill patients			
Yes (n %)	21 (36.2)	19 (37.3)	*χ* ^2^ 0.13
No (n %)	37 (63.8)	32 (62.7)	0.910
Experience with simulation			
Yes (n %)	6 (10)	5 (9.6)	*χ* ^2^ 0.005
No (n %)	54 (90)	47 (90.4)	0.946

^a^t-test.

## Data Availability

The underlying data will be available through the USN Research Data Archive, DOI 10.23642/usn.6148562.
